# Supplementary material for: Uniparental nuclear inheritance following bisexual mating in fungi
Source: eLife. 2021 Aug 2;10:e66234. doi: 10.7554/eLife.66234 (PMC8412948; doi:10.7554/eLife.66234)
Supplement: Supplementary file 1. — (a). The genotype of basidia-specific spores dissected from H99α×Bt63a and VYD135α×Bt63a crosses. (b). The genotype of basidia-specific spores dissected from H99α×IUM96-2828a and VYD135α×IUM96-2828a crosses. (c). Genotype analysis of basidia-specific progeny from H99α dmc1Δ×KN99a dmc1Δ and VYD135α dmc1Δ×KN99a dmc1Δ crosses. (d). Strains used in this study. (e). Primers used in this study. [file elife-66234-supp1.docx]

**Supplementary Information**

for

**Uniparental nuclear inheritance following bisexual mating in fungi**

Vikas Yadav, Sheng Sun, and Joseph Heitman

Department of Molecular Genetics and Microbiology, Duke University Medical Center, Durham, NC 27710, USA

**Supplementary File 1a. The genotype of basidia-specific spores dissected from H99α x Bt63a and VYD135α x Bt63a crosses.**

|  | **Basidia #** | **Spores dissected** | **Spores germinated** | **% germinated** | ***MAT*** | **Mitochondria** |
| --- | --- | --- | --- | --- | --- | --- |
| **H99α x Bt63a cross**  **(2 weeks old)** | 1 | 14 | 2 | 14 | 1**a** + 1**a**/α | **a** |
|  | 2 | 14 | 5 | 36 | 3α + 1**a** + 1**a**/α | **a** |
|  | 3 | 14 | 2 | 14 | 1α + 1**a** | **a** |
|  | 4 | 14 | 0 | 0 | NA | NA |
|  | 5 | 14 | 3 | 21 | 1a + 2**a**/α | **a** |
|  | 6 | 14 | 13 | 93 | 4α + 5**a** + 4**a**/α | **a** |
|  | 7 | 14 | 3 | 21 | 2α + 1**a**/α | **a** |
|  | 8 | 9 | 0 | 0 | NA | NA |
|  | 9 | 12 | 3 | 25 | 1α + 2**a** | **a** |
|  | 10 | 22 | 6 | 27 | 5α + 1**a**/α | **a** |
| **VYD135α x Bt63a cross**  **(2 weeks old)** | 1 | 6 | 0 | 0 | NA | NA |
|  | 2 | 14 | 6 | 43 | All α | **a** |
|  | 3 | 13 | 13 | 100 | All α | **a** |
|  | 4 | 26 | 0 | 0 | NA | NA |
|  | 5 | 10 | 7 | 70 | All α | **a** |
|  | 6 | 14 | 0 | 0 | NA | NA |
|  | 7 | 14 | 0 | 0 | NA | NA |
|  | 8 | 14 | 13 | 93 | All α | **a** |
|  | 9 | 20 | 18 | 90 | All α | **a** |
|  | 10 | 16 | 0 | 0 | NA | NA |
| **VYD135α x Bt63a cross**  **(5 weeks old)** | 11 | 14 | 11 | 79 | All α | **a** |
|  | 12 | 14 | 6 | 43 | All α | **a** |
|  | 13 | 28 | 18 | 64 | All α | **a** |
|  | 14 | 14 | 12 | 86 | All α | **a** |
|  | 15 | 7 | 4 | 57 | All α | **a** |
|  | 16 | 12 | 9 | 75 | All α | **a** |
|  | 17 | 28 | 19 | 68 | All α | **a** |
|  | 18 | 22 | 6 | 27 | All α | **a** |
|  | 19 | 14 | 7 | 50 | All α | **a** |
|  | 20 | 12 | 12 | 100 | All α | **a** |

NA – Not Applicable

**Supplementary File 1b. The genotype of basidia-specific spores dissected from H99α x IUM96-2828a and VYD135α x IUM96-2828a crosses.**

|  | **Basidia #** | **Spores dissected** | **Spores germinated** | **% germinated** | ***MAT*** | **Mitochondria** |
| --- | --- | --- | --- | --- | --- | --- |
| **H99α x IUM96-2828a cross**  **(2 weeks old)** | 1 | 26 | 21 | 81 | 10α + 11**a** | **a** |
|  | 2 | 28 | 28 | 100 | 16α + 12**a** | **a** |
|  | 3 | 14 | 9 | 64 | 6α + 3**a** | **a** |
|  | 4 | 20 | 6 | 30 | 4α + 1**a** + 1**a**/α | **a** |
|  | 5 | 22 | 0 | 0 | NA | NA |
|  | 6 | 15 | 0 | 0 | NA | NA |
| **H99α x IUM96-2828a cross**  **(5 weeks old)** | 7 | 19 | 17 | 89 | All α | **a** |
|  | 8 | 20 | 20 | 100 | All α | **a** |
|  | 9 | 20 | 18 | 90 | All α | **a** |
|  | 10 | 26 | 7 | 27 | **a**/α | **a** |
|  | 11 | 14 | 2 | 14 | All α | **a** |
|  | 12 | 14 | 5 | 36 | 3α + 1**a** + 1**a**/α | **a** |
| **VYD135α x IUM96-2828a cross**  **(2 weeks old)** | 1 | 12 | 0 | 0 | NA | NA |
|  | 2 | 23 | 0 | 0 | NA | NA |
|  | 3 | 14 | 11 | 79 | All α | **a** |
|  | 4 | 13 | 0 | 0 | NA | NA |
|  | 5 | 16 | 3 | 19 | All **a** | **a** |
|  | 6 | 14 | 0 | 0 | NA | NA |
| **VYD135α x IUM96-2828a cross**  **(5 weeks old)** | 7 | 14 | 0 | 0 | All α | **a** |
|  | 8 | 23 | 10 | 43 | All α | **a** |
|  | 9 | 22 | 19 | 86 | All α | **a** |
|  | 10 | 23 | 22 | 96 | All α | **a** |
|  | 11 | 28 | 24 | 86 | All α | **a** |
|  | 12 | 28 | 14 | 50 | All α | **a** |
|  | 13 | 22 | 20 | 91 | All α | α |
|  | 14 | 22 | 22 | 100 | All α | **a** |
|  | 15 | 24 | 13 | 54 | All α | **a** |
|  | 16 | 17 | 8 | 47 | All α | **a** |
|  | 17 | 9 | 8 | 89 | All α | **a** |
|  | 18 | 24 | 9 | 38 | All α | **a** |

NA – Not Applicable

**Supplementary File 1c. Genotype analysis of basidia-specific progeny from H99α *dmc1*Δ x KN99a *dmc1*Δ and VYD135α *dmc1*Δ x KN99a *dmc1*Δ crosses.**

| **Basidia #** | H99α *dmc1*Δ x KN99**a** *dmc1*Δ cross | | | | VYD135α *dmc1*Δ x KN99a *dmc1*Δ cross | | | |
| --- | --- | --- | --- | --- | --- | --- | --- | --- |
|  | **Spores germinated/ dissected** | **% germinated** | ***MAT*** | **Mito** | **Spores germinated/ dissected** | **% germinated** | ***MAT*** | **Mito** |
| 1 | 7/24 | 29 | 2α + 5**a**/α | **a** | 0/26 | 0 | **-** | **-** |
| 2 | 2/20 | 10 | All α | **a** | 7/14 | 50 | All **a** | **a** |
| 3 | 3/20 | 15 | All α | **a** | 0/10 | 0 | **-** | **-** |
| 4 | 5/14 | 36 | All **a**/α | **a** | 8/18 | 44 | All **a** | **a** |
| 5 | 3/11 | 27 | All **a** | **a** | 12/12 | 100 | All **a** | **a** |
| 6 | 0/12 | 0 | - | **-** | 7/8 | 88 | All **a** | **a** |
| 7 | 7/26 | 27 | All **a** | **a** | 14/14 | 100 | All **a** | **a** |
| 8 | 0/9 | 0 | - | **-** | 0/8 | 0 | **-** | **-** |
| 9 | 0/19 | 0 | - | **-** | 0/14 | 0 | **-** | **-** |
| 10 | 1/11 | 9 | α | **a** | 19/19 | 100 | All **a** | **a** |
| 11 | 24/27 | 89 | 12α + 6**a** + 6**a**/α | **a** | 0/5 | 0 | **-** | **-** |
| 12 | 5/22 | 23 | 1α + 4**a**/α | **a** | - | - | **-** | **-** |

Mito refers to Mitochondria.

**Supplementary File 1d. Strains used in this study.**

| Strain name | Description | Reference |
| --- | --- | --- |
| H99 | Wild-type *MAT*α | (Perfect, Ketabchi, Cox, Ingram, & Beiser, 1993) |
| KN99**a** | Wild-type *MAT***a** | (Nielsen et al., 2003) |
| VYD135 | H99 derivative with shuffled chromosomes | (Yadav, Sun, Coelho, & Heitman, 2020) |
| Bt63 | Wild-type *MAT***a** | (Litvintseva et al., 2003) |
| IUM96-2828 | Wild-type *MAT***a** | (Keller, Viviani, Esposto, Cogliati, & Wickes, 2003) |
| VYD158 | H99α *GFP-H4*::*NAT* | This study |
| VYD159 | VYD135α *GFP-H4*::*NAT* | This study |
| VYD160 | KN99**a** *mCherry-H4*::*NAT* | This study |
| VYD171 | Bt63**a** *mCherry-H4*::*NAT* | This study |
| VYD178 | H99α *dmc1*Δ::*NEO* | This study |
| VYD179 | KN99a *dmc1*Δ::*NEO* | This study |
| VYD181 | VYD135 *dmc1*Δ::*NEO* | This study |
| VYD182 | KN99**a** *mCherry-H4*::*NAT* *dmc1*Δ::*NEO* | This study |

**Supplementary File 1e. Primers used in this study.**

| **Primer #** | **Sequence** | **Purpose** |
| --- | --- | --- |
| JOHE50979 | CTAACTCTACTACACCTCACGGCA | *MAT***a** PCR |
| JOHE50980 | CGCACTGCAAAATAGATAAGTCTG |  |
| JOHE50981 | GGCTGCAATCACAGCACCTTAC | *MAT*α PCR |
| JOHE50982 | CTTCATGACATCACTCCCCTAT |  |
| JOHE51004 | TGGTGGTGGTGACCCAGTTCT | Mitochondria genotyping primers for H99, VYD135, KN99, IUM96 |
| JOHE51005 | CCGAAGATCTTAGGTGCCCA |  |
| JOHE51006 | CCACAACCTATTAACATTAGCTACGC | Mitochondria genotyping primers for H99, VYD135, Bt63 |
| JOHE51007 | CGTCTCCATCTACAAAGCCAGCAAAC |  |
| JOHE51159 | ACCGGCAGGGTATACTGTTGAGTGCTGTGGTGAAAGAGATGTTTTAGAGCTAGAAATAGC | Safe Haven guide RNA |
| JOHE51204 | GCATGCGAGCTCGGCAGATACGATATGTTGGCG | P_H3_-mCherry contruct |
| JOHE51205 | CCTCCTCGCCCTTGCTCACCATTGATAGATGTGTTGTGGTG |  |
| JOHE51206 | CACCACAACACATCTATCAATGGTGAGCAAGGGCGAGGAGG |  |
| JOHE51207 | GCATGCGGATCCCTTGTACAGCTCGTCCATGCC |  |
| JOHE51403 | GTCCAAAGTACTCAATGATACGACC | Dmc1 deletion construct |
| JOHE51404 | CAGCTCACATCCTCGCAGCCATAGGAAGGGTGGCAAATCCAAATGAC |  |
| JOHE51405 | GTCATTTGGATTTGCCACCCTTCCTATGGCTGCGAGGATGTGAGCTG |  |
| JOHE51406 | GTCGAGTCAAAATGAATAGGAATCCGCGGTTTATCTGTATTAACACGG |  |
| JOHE51407 | CCGTGTTAATACAGATAAACCGCGGATTCCTATTCATTTTGACTCGAC |  |
| JOHE51408 | AATTGTGGAACAAGAACGAGGAC |  |
| JOHE51409 | TAGCCAACATACCCAAACCACCAGC |  |
| JOHE51410 | GTGGTTGTTTGAGTCGTTTGAGAGC |  |
| JOHE51416 | ACCGGCAGGGTATACTGTTGATAATAGGCCGGCTTCTGGTGTTTTAGAGCTAGAAATAGC | Dmc1 deletion guide RNA |
| JOHE51417 | ACCGGCAGGGTATACTGTTGGATTCACCTGATATGCCGGAGTTTTAGAGCTAGAAATAGC |  |
